# Blue light at night acutely impairs glucose tolerance and increases sugar intake in the diurnal rodent *Arvicanthis ansorgei* in a sex‐dependent manner

**DOI:** 10.14814/phy2.14257

**Published:** 2019-10-23

**Authors:** Anayanci Masís‐Vargas, David Hicks, Andries Kalsbeek, Jorge Mendoza

**Affiliations:** ^1^ Institute of Cellular and Integrative Neurosciences (INCI) UPR‐3212 CNRS University of Strasbourg Strasbourg France; ^2^ Hypothalamic Integration Mechanisms Netherlands Institute for Neuroscience (NIN) Amsterdam The Netherlands; ^3^ Department of Endocrinology and Metabolism Amsterdam UMC University of Amsterdam Amsterdam The Netherlands

**Keywords:** *Arvicanthis ansorgei*, Blue light, circadian clock, food intake, glucose intolerance, high‐fat high‐sugar diet, insulin sensitivity

## Abstract

In our modern society, the exposure to light at night (LAN) has increased considerably, which may impact human health negatively. Especially exposure to light at night containing short wavelength emissions (~450–500 nm) can disrupt the normal function of the biological clock, altering sleep‐wake cycles and inducing metabolic changes. Recently, we reported that light at night acutely impairs glucose tolerance in nocturnal rats. However, light at night in nocturnal rodents coincides with their activity period, in contrast to artificial light at night exposure in humans. The aim of this study was to evaluate the acute effects of blue (*λ* = 490 ± 20 nm) artificial light at night (bALAN) on glucose metabolism and food intake in both male and female diurnal Sudanian grass rats (*Arvicanthis ansorgei*) fed either regular chow or a free choice high‐fat high sucrose diet (HFHS). In both chow and HFHS fed male *Arvicanthis*, 1‐hour of bALAN exposure induced a higher glucose response in the oral glucose tolerance test (OGTT) accompanied by a significant decrease in plasma insulin. Furthermore, in HFHS fed animals, bALAN induced an increase in sucrose intake during the dark phase in males but not in females. Additionally, 1‐h of bALAN increased the nonfasted glucose levels together with plasma corticosterone in female grass rats. These results provide new and further evidence for the deleterious effects of exposure to short wavelength emission‐containing artificial light at night on glucose metabolism in a diurnal rodent in a sex‐dependent manner.

## Introduction

Data from the World Health Organization show that the prevalence of obesity has increased almost three times since 1975. By 2016 more than 1.9 billion adults were overweight, of which 650 million were obese. Nowadays in most countries, obesity accounts for more deaths than underweight (WHO 2017b). It is widely known that obesity is a major risk factor for noncommunicable diseases such as type 2 diabetes. The number of people suffering from type 2 diabetes has risen from 108 million in 1980 to 422 million in 2014 (WHO 2017a). The economic burden for healthcare systems associated with the diagnosis and health care of diabetic patients is increasing profoundly. Type 2 diabetes as much as obesity are multifactorial diseases with complex underlying physio‐pathological mechanisms, nevertheless for both diseases many cases are preventable through lifestyle changes.

Epidemiological and experimental studies in humans have shown that sleep disruption and exposure to artificial light at night (ALAN) are risks factors for the development of obesity (Obayashi et al. 2013; McFadden et al. 2014) and type 2 diabetes (Chaput et al. 2009; St‐Onge et al. 2012; Obayashi et al. 2014). Furthermore, animal studies have shown that exposure to ALAN increases body mass (Fonken et al. 2013a; Borniger et al. 2014; Aubrecht et al. 2015; Cho et al. 2015; Cissé et al., 2017), exacerbates inflammatory responses (Fonken et al. 2013, 2013a,b), alters food intake (Cissé et al. 2017), disrupts metabolism and circadian rhythms (Borniger et al. 2014; Kayaba et al. 2014), and changes insulin sensitivity (Coomans et al. 2013).

The strong relationship between circadian rhythms and metabolism is now well established, especially regarding the control of glucose homeostasis (La Fleur et al. 2001, Cailotto et al. 2005). Although most endogenous rhythms will continue to oscillate even without the presence of an environmental light/dark cycle; light is the most important environmental signal to entrain the intrinsic ~24 h (i.e., circadian) rhythms to the 24‐h rhythm of the rotation of the Earth. However, light can also be a potent circadian and endocrine disruptor when received at the wrong time of the day, that is, during the dark phase (Russart and Nelson 2018), causing alterations in the secretion of the hormones melatonin (Kalsbeek et al. 1999) and corticosterone (cortisol in humans) (Ishida et al. 2005), and changes in the expression of circadian genes in the hypothalamus (Best et al. 1999), pineal gland (Wu et al. 2008), adrenal (Ishida et al. 2005), and liver (Cailotto et al. 2009), all of them strongly involved in glucose metabolism.

In mammals, light is perceived by rod and cone photoreceptors in the retina eliciting both visual and non‐image forming visual responses. Previous studies have revealed the existence of intrinsically photosensitive retinal ganglion cells (ipRGCs) that express the photopigment melanopsin (with a peak spectrum of absorption at *λ* = 480 nm, i.e., blue light), in addition to rods and cones. The anatomical projections of the ipRGCs directly reach the hypothalamic suprachiasmatic nucleus (SCN), the site of the main circadian clock, and other parts of the brain involved in energy metabolism, glucose homeostasis, reward and food intake (Provencio et al. 2000; Hattar et al. 2006; LeGates et al. 2014). Combined with the evidence that ALAN is a risk factor for metabolic diseases, this leads to the hypothesis that ALAN can alter food intake and glucose metabolism.

In a previous study from our group, we showed that exposure to ALAN in male rats acutely induced glucose intolerance, most likely by reducing beta cell sensitivity and glucose uptake (Opperhuizen et al. 2017). However, the nocturnal nature of rats and the metabolic differences between sexes (Goel et al. 2014) is an important and complicating factor to consider when translating these findings to the human situation. Therefore, in this study, we used a diurnal rodent to study the acute effects of ALAN on food intake and glucose metabolism in both males and females. In addition, we used a palatable diet and blue ALAN (bALAN), which both are widely present in human daily modern life.

## Materials and Methods

### Ethical approval

All the experiments were performed in accordance with the rules of the European Committee Council Directive of November 24, 1986 (86/609/EEC) and the French Department of Agriculture (license no. 63‐378 to JM). The investigators understand the ethical principles under which the journal operates and all experiments comply with the policies and regulations set out in the editorial (Grundy 2015).

### Animals and housing

Thirty‐five 12‐weeks‐old male (*n* = 18) and female (*n* = 17) Sudanian grass rats (*Arvicanthis ansorgei*) obtained from the platform Chronobiotron (UMS‐3415, CNRS and University of Strasbourg) weighing 131 ± 17 g were individually housed in Plexiglas cages with sawdust bedding, chow food and tap water ad libitum. Animals were maintained under a controlled ambient temperature of 21–23°C, a relative humidity of 23–27% and a 12‐h light/dark (LD) cycle (lights on at 07:00 h, *Zeitgeber* time 0 (ZT0); lights off 19:00 h, ZT12) with dim red light at night (5 lux).

### Daily food intake rhythm

**Figure 1:**
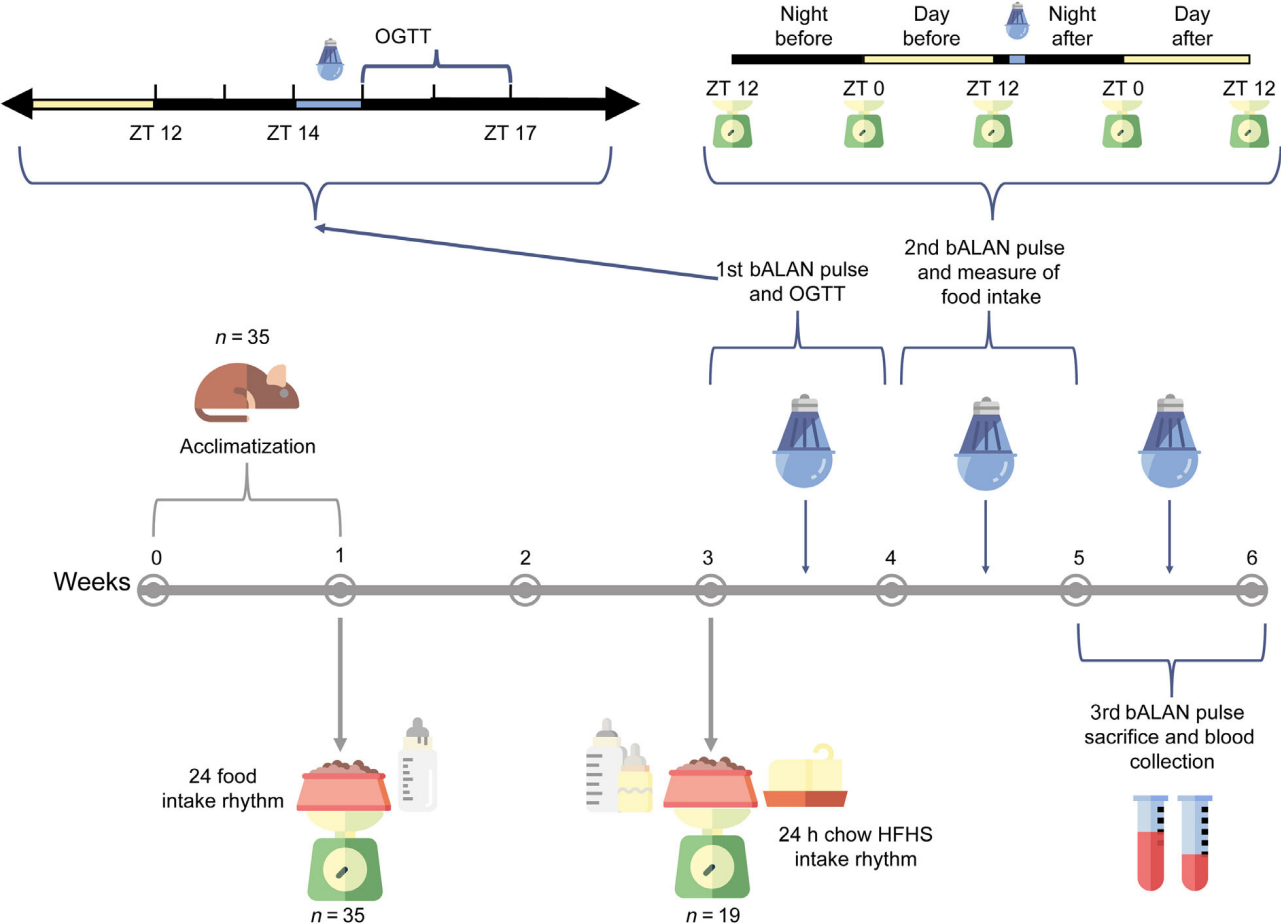
Graphical representation of the experimental design.

Chow food (SAFE, 105, U8400G10R, Augy, France; 2.85 kcal/g, 23% proteins, 65% carbohydrates, and 12% fat) and tap water were provided ad libitum to all animals. After a week of acclimatization, food consumption was measured manually every 4 h for 4 days, to assess the daily food intake pattern (Fig. 1).

### Free choice high‐fat high‐sucrose diet

Immediately after measuring the daily food intake rhythm with chow food, animals were randomly assigned to two groups fed with different diets. The control group (*n* = 8 per sex) remained on the chow diet mentioned before and tap water. The free choice High‐Fat High‐Sugar (HFHS) group (*n* = 10 males and *n* = 9 females) received in addition to the chow food and the tap water, a cube of saturated fat (beef lard, Vandemoortele, France; 9 kcal/g) and a bottle of 10% sugar water (commercial grade sucrose and tap water, 0.4 kcal/mL). In the HFHS model, animals are free to choose what they prefer and are not forced to eat a high caloric diet as in other rodent models. This situation reflects human conditions in which people can choose from different diets (normocaloric vs. hypercaloric). After two weeks on the HFHS diet food consumption was again measured manually every 4 h for 4 days, as described before, to assess the daily food intake rhythm when animals were fed with the HFHS diet. Both groups were kept on their respective diets for 4 more weeks and animals were weighed weekly (Fig. 1).

### Light exposure protocol

To study whether the exposure to blue artificial light at night (bALAN) has an effect on glucose metabolism and food intake, starting in week 3 animals from both diet groups were randomly sub‐divided in two subgroups, dark controls (*n* = 9 chow‐fed, 4 males and 5 females; *n* = 10 HFHS‐fed, 6 males and 4 females) and blue light exposed (*n* = 7 chow‐fed, 4 males and 3 females; *n* = 9 HFHS‐fed, 4 males and 5 females) (Fig. 1). At ZT14 (2 h after lights off) individual cages of the animals from the light group were moved to a PVC cage (120 × 60 × 60 cm), which had a fixed blue light‐emitting diode (LED) lamp placed in the middle of the ceiling and they were exposed to a 1‐h pulse of blue light (490 ± 20 nm wavelength, see Table 1 for the irradiance spectrum (Lucas et al. 2014)). Then, the dark condition was restored and animals from the light group were moved back to their racks. Protocols to evaluate glucose metabolism, food intake and light effects on hormones were applied immediately thereafter as described below. Between each light exposure, animals were left undisturbed for 1 week.

**Table 1:** Spectral sensitivity of the light condition used in the whole experiments.

Retinal photopigment complement	Blue light
S‐Cone (rN_sc_ [λ])	0.00
Melanopsin (rN_z_ [*λ*])	27.20
Rod (rN_z_ [*λ*])	26.55
M‐cone (rN_mc_ [*λ*])	24.34
Irradiance (*µ*W/cm^2^)	4.26
Photon flux (1 cm^−2^ sec^−1^)	1.06 × 10^13^

Data based on the Rodent Toolbox provided by Lucas et al (2014).

The first four rows represent weighted contribution of rodent retinal photopigments (S‐cone, ipRGC [melanopsin], rod, M‐cone) in α‐opic rodent lux. The last two rows (irradiance, photon flux) represent unweighted characteristics of the LEDs.

### Oral glucose tolerance test (OGTT)

Immediately after animals were returned from the exposure to either the first pulse of blue light or being kept in control dark conditions respectively, an OGTT was performed starting at ZT15 (Fig. 1). For this, all *Arvicanthis* were fasted for 6 h before the test, that is, from ZT9 onwards. Blood was taken from the tip of the tail at 0, 15, 30, 90, and 120 min for the measurement of whole blood glucose concentrations using an Accu‐Chek Performa Nano glucometer (Roche Diabetes Care Limited). Immediately after the 0 min sample, d‐glucose (2g of d‐glucose per kg of body weight dissolved in 0.9% saline) was orally administered by gavage. Data of the OGTT were expressed as the delta between the basal (*t* = 0) glucose and the measurement at each time point. In addition, the increase of plasma glucose was analyzed by calculating the area under the curve (AUC) from the time of the baseline to the 120 min measurement, using the trapezoidal rule (Allison et al. 1995).

### Food intake evaluation

One week after the OGTT, animals received the same light treatment described above for the second time. All food components from both diet groups were weighed every 12 h during 2 LD cycles, starting at ZT12 (the onset of dark phase) before light exposure, until ZT12 (the off of light phase) after, in order to measure possible changes in the daily food intake patterns due to the blue light pulse at the beginning of the dark phase.

### Locomotor activity recordings

To measure activity‐rest cycles, general locomotor activity of all animals was monitored using infrared detectors placed above the cages linked to an automated recording system (CAMS, Circadian activity monitoring system, Lyon, France). Data were recorded every 5 min. Clocklab software (Actimetrics, Wilmette, IL) was used to determine the activity profiles of each animal under different diet conditions (chow vs. HFHS).

### Blood sampling and serum analysis

On the last week of the experiment, a third light pulse was given, and animals were deeply anesthetized immediately after (i.e., ZT15) with an overdose of isoflurane to recover the blood from the left ventricle with a cardiac puncture, using a 5‐mL syringe with a 25G needle. Immediately after animals were perfused transcardially with PFA 4% wt/vol in 0.1 mol/L phosphate buffer (pH = 7.2) to recover brains for analysis in future studies. Glucose levels in blood were determined as described before using a drop of the extracted blood. Then the blood was decanted into 15 mL Corning tubes containing 100 *µ*L of 4% EDTA. Samples were centrifuged 3913 *g* at 4ºC during 10 min, and plasma was stored at −80°C for determination of plasma insulin, corticosterone and leptin concentrations. Insulin was measured using an ELISA procedure with a Rat/Mouse Insulin ELISA Kit (EZRMI‐13K, Merck‐Millipore, Germany). The limit of sensitivity of the insulin kit was 0.1 ng/mL. Corticosterone was measured using an EIA procedure with a Rat/Mouse Corticosterone EIA Kit (AC‐14F1, Immunodiagnostic Systems Ltd, UK). The limit of sensitivity corticosterone kit was 0.55 ng/mL. The levels of leptin in plasma were measured using an ELISA procedure with a Rat Leptin Kit (EZRL‐83K, Merck‐Millipore, Germany). The limit of sensitivity of the leptin kit was 0.04 ng/mL.

### Statistical analysis

Results were analyzed with and without taking into account the sex of the animals. All data are expressed as means ± standard deviation. A two‐way ANOVA was used to compare locomotor activity and caloric intake between light versus dark phases and between diet groups (chow vs. HFHS), and, in the HFHS group, to compare the intake of the components (chow vs. fat vs. sugar) of the diet at the light versus night phase.

Unpaired two‐tailed *t* tests were used to detect group differences (dark vs. bALAN) on fasted and non‐fasted glucose, the areas under the curve (AUCs), and plasma hormones after the light exposure.

A two‐way mixed model ANOVA was used to analyze the OGTT and food intake data before and after the light pulse. All ANOVA’s results were followed by a Sidak’s multiple comparisons as a post‐hoc test. Statistical power was calculated with a post‐hoc two‐tail *t*‐test (power = 85%, *d* = 2.86, *α* = 0.05) using G Power 3.1.9.2. GraphPad Prism version 7.01 for Windows (GraphPad Software, La Jolla, CA, USA) and IBM SPSS Statistics for Windows version 25 (Armonk, NY: IBM Corp.) using a significance level of *P* < 0.05.

## Results

### Locomotor activity and daily food intake rhythms

**Figure 2:**
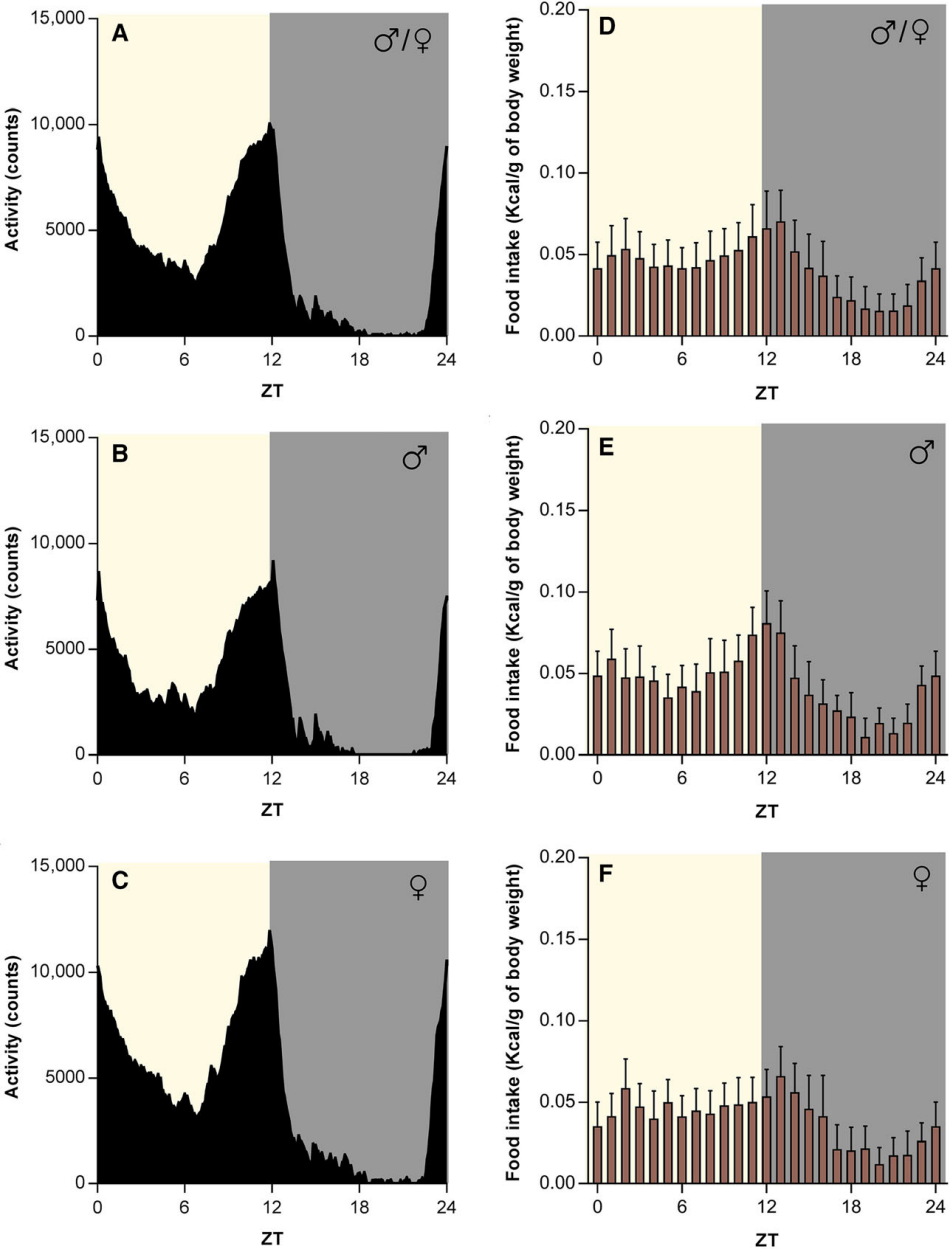
Chow‐fed *Arvicanthis ansorgei* show a biphasic crepuscular locomotor activity and food intake pattern. The plot of the mean 24‐h locomotor activity of both sex shows a biphasic crepuscular shape (A), with similar behavior in male (B) and female (C) grass rats when fed a chow diet. The biphasic activity pattern correlates with the food intake rhythm in both sexes together (D). The two phases of the food intake, i.e., dusk and dawn, are observable in males (E) and females (F). Food intake is expressed as kilocalories consumed per gram of body weight. ZT, *Zeitgeber* Time (in hours).

Both male and female *Arvicanthis* fed with the chow diet showed stable and significant diurnal activity with a crepuscular pattern, with a higher locomotion before lights on and off, and continued stable during the light phase (*F*_(288,4032)_ = 33.1, *P* < 0.001; Fig. 2A–C). The daily food intake rhythm of chow‐fed male and female animals showed also a diurnal intake with a biphasic pattern (*F*_(23,1150)_ = 60.4, *P* < 0.001), which fits with the crepuscular pattern of locomotion (Fig. 2D–F).

**Figure 3:**
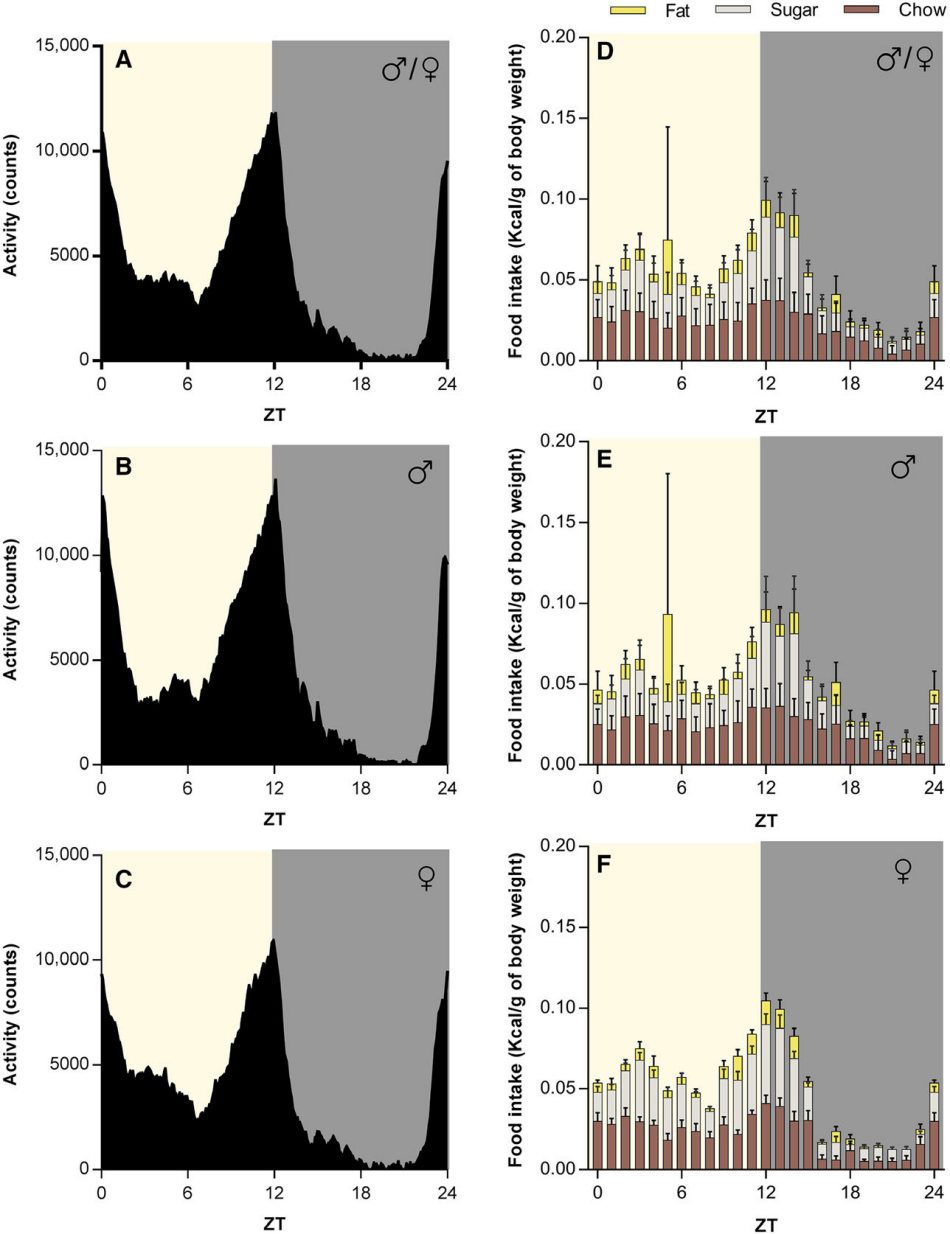
HFHS‐fed *Arvicanthis ansorgei* show a biphasic crepuscular locomotor activity and food intake pattern. The plot of the mean 24‐hour locomotor activity of both sexes (A) shows a biphasic crepuscular shape. This activity pattern when separated is similar between male (B) and female grass rats (C) fed with a HFHS diet. The biphasic activity pattern correlates with the HFHS food intake rhythm in both sexes together (D). Food intake patterns of the different components of the HFHS diet (fat, sugar, chow) in both males (E) and females (F). Food intake is expressed as kilocalories consumed per gram of body weight. ZT, *Zeitgeber* Time (in hours).

The crepuscular pattern of locomotor activity and food intake was also observed when animals from both sexes were fed with a HFHS diet (locomotion, *F*_(288,4896)_ = 33.4, *P* < 0.001; Feeding, *F*_(23,1242)_ = 16.9, *P* < 0.001; Fig. 3A–F). When we compare the daily rhythms of food intake for each component of the HFHS diet, we found significant differences in the consumption of the chow (*F*_(23,391)_ = 1.8, *P* = 0.01) and fat (*F*_(23,391)_ = 3.4, *P* < 0.001) by sex; being higher in males at ZT17 (Post‐hoc, *P* = 0.006) and ZT5 (Post‐hoc, *P* < 0.001) respectively (Fig. 3E and F).

**Figure 4:**
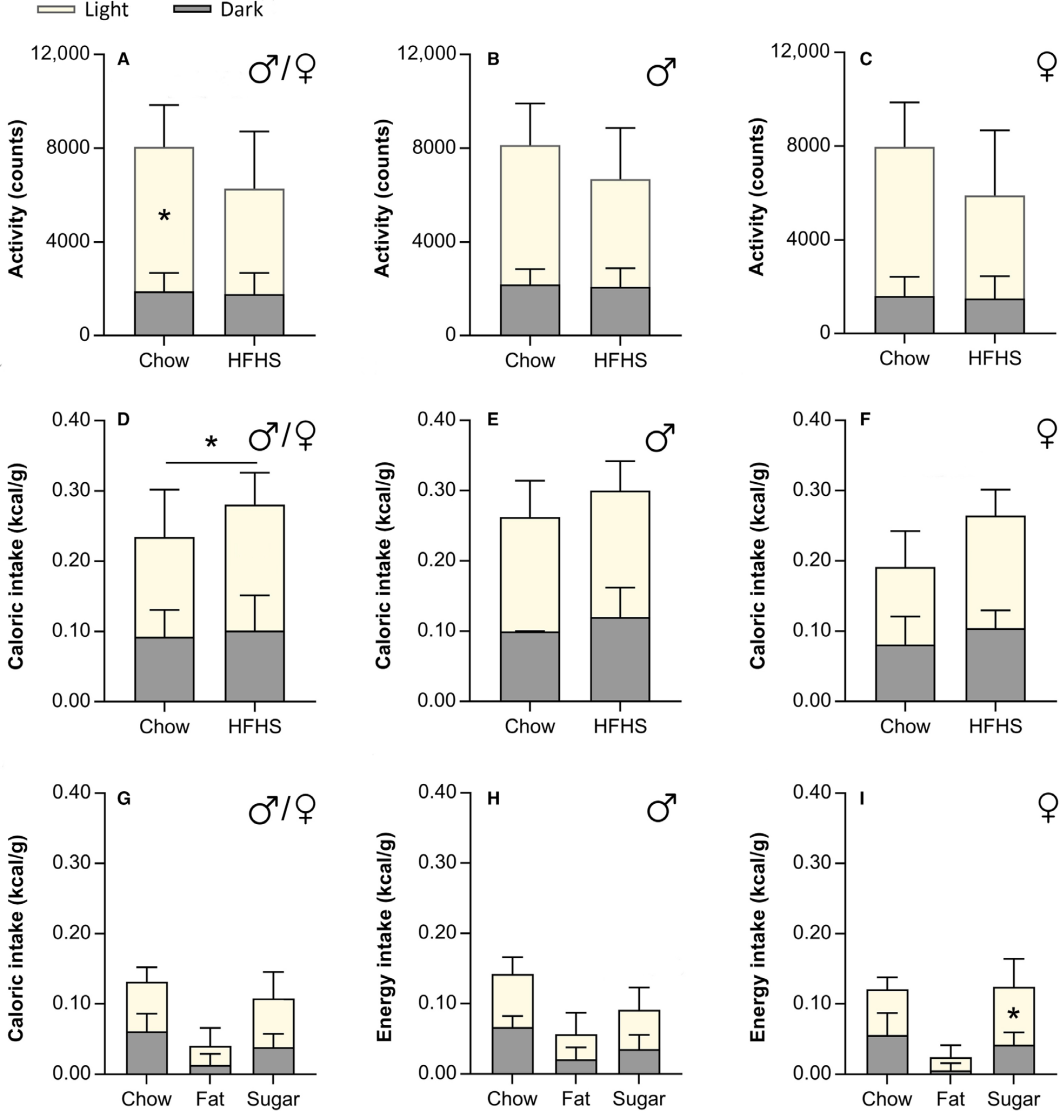
HFHS diet induces changes in the total caloric intake and locomotor activity of *Arvicanthis ansorgei.* Light versus dark phase activity in males and females *Arvicanthis* together (A) or separated by sex (B and C) fed with a chow or a HFHS diet. Activity was significantly higher at the light phase in both males and females. Moreover activity at the light phase in both sexes together (A) was significant higher in chow‐fed than HFHS‐fed animals (*Post‐hoc, *P* = 0.008). (D) Animals of both sexes from either diet group ate more total calories at the light phase than night. HFHS‐fed animals ingest significantly more calories than the chow group (*Post‐hoc, *P* = 0.009). Separately, in males (E) and females (F) grass rats caloric intake is higher at the light phase, but no differences in the total caloric intake between diet groups (chow vs. HFHS) were found. Intake at the light versus dark phase of the different components of the HFHS diet from both sexes together (G), or separately in males (H) and females (I). Female *Arvicanthis* drink significantly more sugar during the light phase than the dark phase (*Post‐hoc, *P* = 0.0001).

In the analysis of males and females *Arvicanthis* together, the total 24 h locomotor activity was not different between diet groups (*F*_(1,33)_ = 3.9, *P* = 0.05; Fig. 4A). However, a significant difference in locomotion for the factor phase (light vs. dark phase, *F*_(1,33)_ = 108.7, *P* < 0.001), and for the interaction between phase X diet factors was found (*F*_(1,33)_= 5.2, *P* = 0.029), indicating that activity during the light phase was higher in chow‐fed than HFHS‐fed *Arvicanthis* (Post‐hoc, *P* = 0.008; Fig. 4A)*.* When analyzed by sex, only a significant difference in the levels of locomotor activity for the factor phase (light vs. dark), but not for the factor diet or the interaction phase X diet, was found in both males (*F*_(1,15)_ = 38.6, *P* < 0.0001) and females (*F*_(1,16)_ = 70.7, *P* < 0.0001), with a highest activity at the light phase (Fig. 4B–C).

Caloric intake was higher at the light phase in both diet groups (Chow and HFHS) (*F*_(1,33)_ = 30.8, *P* < 0.0001; Fig. 4D). However, the 24 h total (light and dark phase) energy consumption was significantly higher in HFHS‐fed animals (*F*_(1,33)_ = 7.5, *P* = 0.009; Fig. 4D). Separately, both males (*F*_(1,16)_ = 20.8, *P* < 0.0001) and females (*F*_(1,15)_ = 39.2, *P* = <0.0001) ate significantly more during the light phase (Fig. 4E and F). In HFHS‐fed females, but not males, the 24 h total caloric intake was higher compared with chow‐fed female *Arvicanthis*, although this effect was at the limit of significance (*F*_(1,15)_ = 4.25, *P* = 0.05; Fig. 4F).

In HFHS‐fed animals, when we analyzed calorie intake together in males and females *Arvicanthis* (Fig. 4G), the two‐way ANOVA indicated a light versus dark phase difference (*F*_(2,102)_ = 13.8, *P* = 0.0003) and in the intake of the three components of the HFHS diet (chow vs. fat vs. sugar; *F*_(2,102)_ = 32.5, *P* < 0.001), but no for the factors interaction (*F*_(2,102)_ = 1.7, *P* = 0.18). However, when analyzed by sex, the intake of the three components of the diet (chow vs. fat vs. sugar) was significantly different between the light versus dark phase in females *Arvicanthis* (time × component interaction, *F*_(2,24)_ = 4.1, *P* = 0.02)**,** showing a main sugar intake at the light phase (Fig. 4I).

Although all animals increased their body weight during the study, with a difference between males and females, no significant differences were observed in body weight gain by light or diet exposure (Table 2).

**Table 2:** Statistical analysis (three‐way ANOVA) of percentage of body weight (BW) gain of the animals during the experiment per diet (Chow vs. HFHS), light treatment (dark vs. bALAN), and sex (male vs. female). Data represent the mean values ± the standard deviation.

	Initial BW (g)	Final BW (g)	BW gain (%)	*P* value for diet	*P* value for light	*P* value for sex
Chow				0.25	0.78	0.02
**♂/♀**	129.3 ± 16.4	133.2 ± 18.2	1.9 ± 7.5			
**♂**	136.2 ± 17.7	146.5 ± 5.9	4.7 ± 3.3			
**♀**	122.4 ± 12.4	120 ± 5.3	−0.8 ± 9.7			
HFHS						
**♂/♀**	131.1 ± 16.9	139.2 ± 19.8	4.2 ± 7.0			
**♂**	138.6 ± 15.9	150.7 ± 16.3	4.6 ± 2.1			
**♀**	122.7 ± 14.5	126.5 ± 15.4	3.8 ± 10.2			

### Acute effects of blue light in glucose metabolism

**Figure 5:**
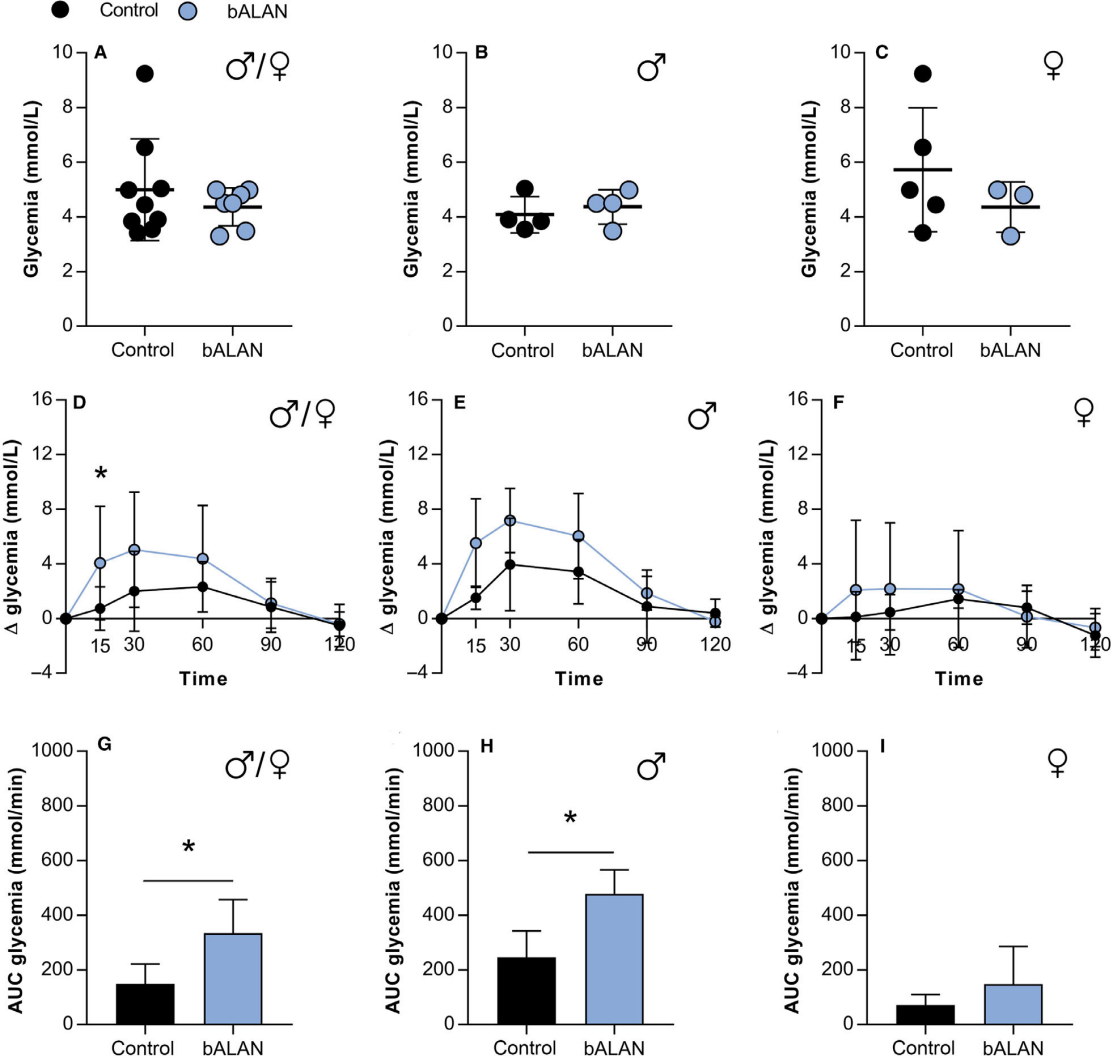
In chow‐fed *Arvicanthis*, baseline concentrations of glucose were not affected by bALAN in all animals (A), nor males (B) or females (C). bALAN exposure from ZT14‐15 in fasted grass rats affects glucose tolerance in all animals after 15 min of the glucose bolus (D; *Post‐hoc, *P* = 0.04) and increases the AUC values of glucose (G; *t‐test*, P* = 0.002). The bALAN exposure altered the glucose response in male *Arvicanthis* (E) by increasing the AUC values of glucose (H; *t‐test*, P* = 0.01). In females no significant change was observed in the glucose response (F) nor in the AUC (I) between control (dark‐exposed) and bALAN‐exposed animals.

**Figure 6:**
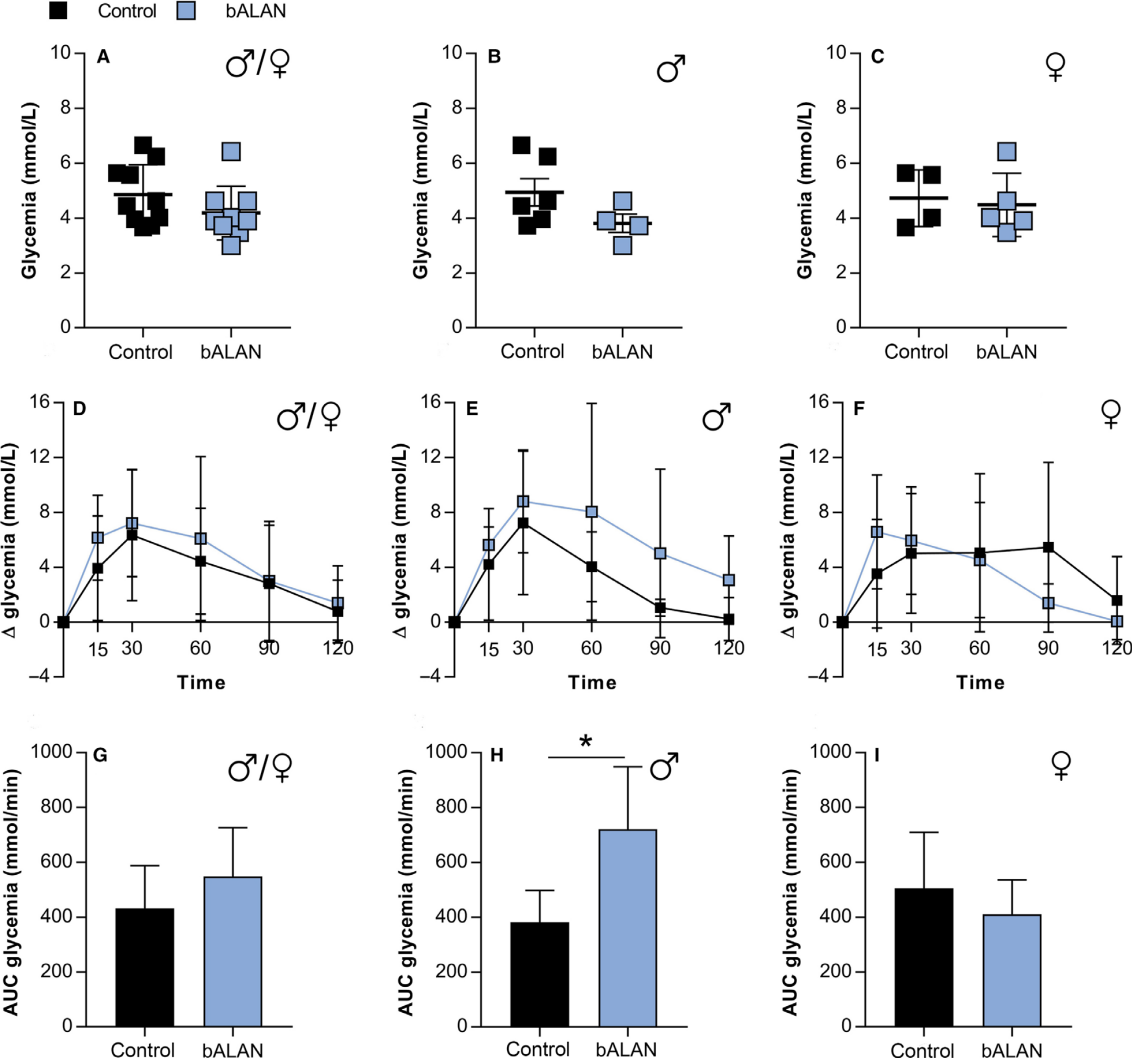
In HFHS‐fed *Arvicanthis* baseline concentrations of glucose were not affected by bALAN in all animals (A), nor males (B) or females (C). bALAN exposure from ZT14‐15 in fasted grass rats did not affect glucose tolerance in all animals (D) nor significantly increased the AUC values (G). bALAN exposure changed the glucose response in male *Arvicanthis* (E) by increasing the AUC values of glucose (H; *t‐test, *P* = 0.01). In females no significant change was observed in the glucose response (F) or in the AUC (I) between control (dark‐exposed) and bALAN‐exposed animals.

Baseline levels of glucose before starting the OGTT were similar between diet and light groups in both males and females (Figs. 5A–C and 6A–C). In chow‐fed animals (males and females), blue light exposure caused a significant increase in plasma glucose 15 min after the glucose bolus (factor time, *F*_(5,70)_ = 12.5, *P* < 0.001; time × treatment interaction, *F*_(5,70)_ = 2.4, *P* = 0.03; Post‐hoc, *P* = 0.04; Fig. 5D, Table 3), as well as a significant increase in the AUC values (*t*_(14)_ = 3.7, *P* = 0.002; Fig. 5G).

**Table 3:** Statistical analysis (mixed‐model ANOVA) of the raw glucose levels during the OGTT of the animals (males/females) fed with the chow diet and exposed to the light treatment (dark vs. bALAN).

Groups		Time (min)						*P* value treatment	*P* value time	*P* value interaction
		0	15	30	60	90	120			
**♂/♀**	Dark	4.9 ± 1.8	5.7 ± 2.2	7.0 ± 3.0	7.3 ± 2.1	5.8 ± 2.1	4.4 ± 1.0	0.229	<0.0001	0.018
	Blue	4.4 ± 0.7	8.4 ± 3.8	9.4 ± 4.0	8.7 ± 3.7	5.5 ± 1.8	3.9 ± 0.9			
**♂**	Dark	4.1 ± 0.7	5.6 ± 1.2	8.0 ± 3.6	7.5 ± 2.3	4.9 ± 2.6	4.5 ± 0.8	0.031	<0.0001	0.036
	Blue	4.4 ± 0.6	9.9 ± 3.2	11.6 ± 1.9	10.4 ± 2.9	6.2 ± 1.6	4.2 ± 0.9			
**♀**	Dark	5.7 ± 2.3	5.8 ± 3.0	6.2 ± 2.6	7.2 ± 2.4	6.5 ± 1.7	4.5 ± 1.4	0.691	0.047	0.665
	Blue	4.4 ± 0.9	6.5 ± 4.2	6.5 ± 4.7	6.5 ± 3.9	4.5 ± 1.8	3.7 ± 1.1			

Data represent the mean values ± the standard deviation of the glucose measurement (mmol/L) during the test from 0 to 120 min after the bolus of glucose administration.

When analyzed separately by sex, the AUC values were significantly higher in males exposed to bALAN (*t*_(6)_ = 3.5, *P* = 0.01; Fig. 5H), whereas no significant difference was found in females (*t*_(6)_ = 1.2, *P* = 0.2; Fig. 5I).

In HFHS‐fed animals, there were no differences between dark versus bALAN exposed‐animals in plasma glucose in the OGTT (*F*_(5,85)_ = 0.3, *P* = 0.8, Table 4) or in AUC glycemia values (*t*_(17)_ = 1.5, *P* = 0.1; Fig. 6D and G). However, when separated by sex, males showed a significant increase in AUC values after bALAN exposure (*t*_(8)_ = 3.1, *P* = 0.01; Fig. 6H), whereas in females no differences were observed between conditions (*t*_(7)_ = 0.8, *P* = 0.4; Fig. 6I). These results indicated a sex‐dependent effect of blue light on glucose metabolism.

**Table 4:** Statistical analysis (mixed model ANOVA) of the raw glucose levels during the OGTT of the animals (males/females) fed with the HFHS diet and exposed to the light treatment (dark vs. bALAN).

Groups		Time (min)						*P* value treatment	*P* value time	*P* value interaction
		0	15	30	60	90	120			
**♂/♀**	Dark	4.9 ± 1.1	8.8 ± 3.5	11.2 ± 4.9	9.3 ± 4.3	7.7 ± 4.7	5.6 ± 2.2	0.05	<0.0001	0.873
	Blue	4.2 ± 0.9	10.3 ± 2.9	11.4 ± 3.8	10.3 ± 6.1	7.2 ± 4.5	5.6 ± 2.7			
**♂**	Dark	4.9 ± 1.2	9.1 ± 3.4	12.2 ± 5.1	9.0 ± 2.8	6.0 ± 0.8	5.2 ± 1.2	0.506	<0.0001	0.651
	Blue	3.8 ± 0.7	9.4 ± 1.4	12.6 ± 4.3	11.9 ± 8.3	8.4 ± 6.7	6.9 ± 3.8			
**♀**	Dark	4.7 ± 1.0	8.3 ± 4.1	9.7 ± 4.9	9.8 ± 6.4	10.2 ± 7.2	6.3 ± 3.4	0.118	0.858	0.100
	Blue	4.5 ± 1.2	11.1 ± 3.7	10.4 ± 3.6	9.0 ± 4.2	5.9 ± 1.5	4.5 ± 0.7			

Data represent the mean values ± the standard deviation of the glucose measurement (mmol/L) during the test from 0 to 120 min after the bolus of glucose administration.

### Acute effects of blue light on food intake

**Figure 7:**
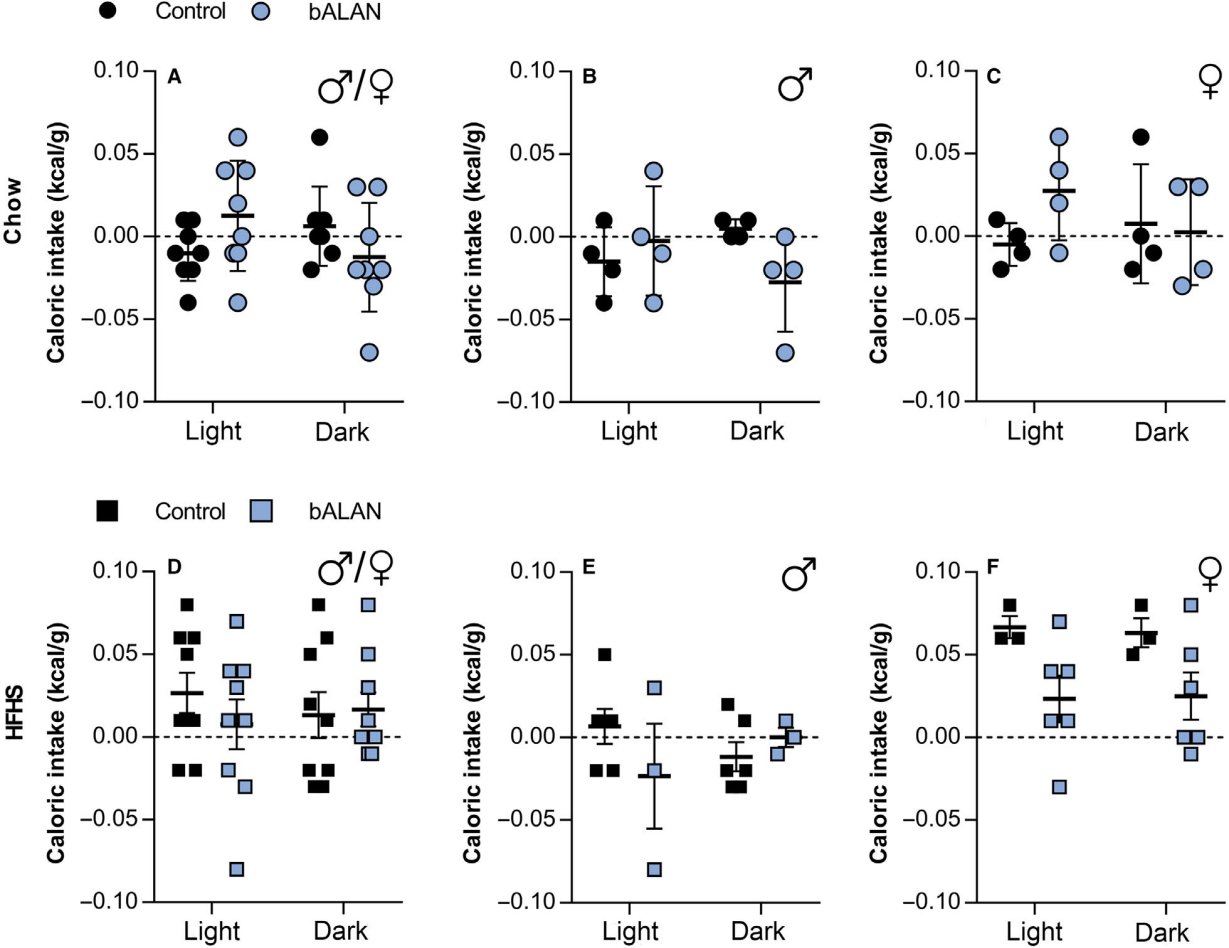
bALAN exposure did not change the total calorie intake in either diet regardless of sex and phase (light vs. dark). bALAN exposure from ZT14‐15 did not affect the calorie intake in all chow‐fed animals (A) nor in males (B) neither in females (C). The bALAN exposure did not affect either the total caloric intake of all animals fed with a HFHS diet (D), neither separately in males (E) or females (F).

We next determined whether acute exposure to bALAN would change food intake. In Figure 7 food intake is depicted as the difference in food consumption between the light versus dark phases before and after the bALAN exposure. When analyzing the total amount of kilocalories per gram of body weight, no significant effects of bALAN exposure were found in the chow‐fed (*F*_(1,14)_ = 0.5, *P* = 0.4) or HFHS‐fed (*F*_(1,16)_ = 0.2, *P* = 0.6) groups in both sexes together (Figs. 7A and D), neither when analyzed separately (chow‐fed males, *F*_(1,6)_ = 1.7, *P* = 0.2, HFHS‐fed males *F*_(1,7)_ = 0.3, *P* = 0.5; chow‐fed females, *F*_(1,6)_ = 0.9, *P* = 0.3, HFHS‐fed females *F*_(1,7)_ = 4.8, *P* = 0.06; Fig. 7B–C and E–F).

**Figure 8:**
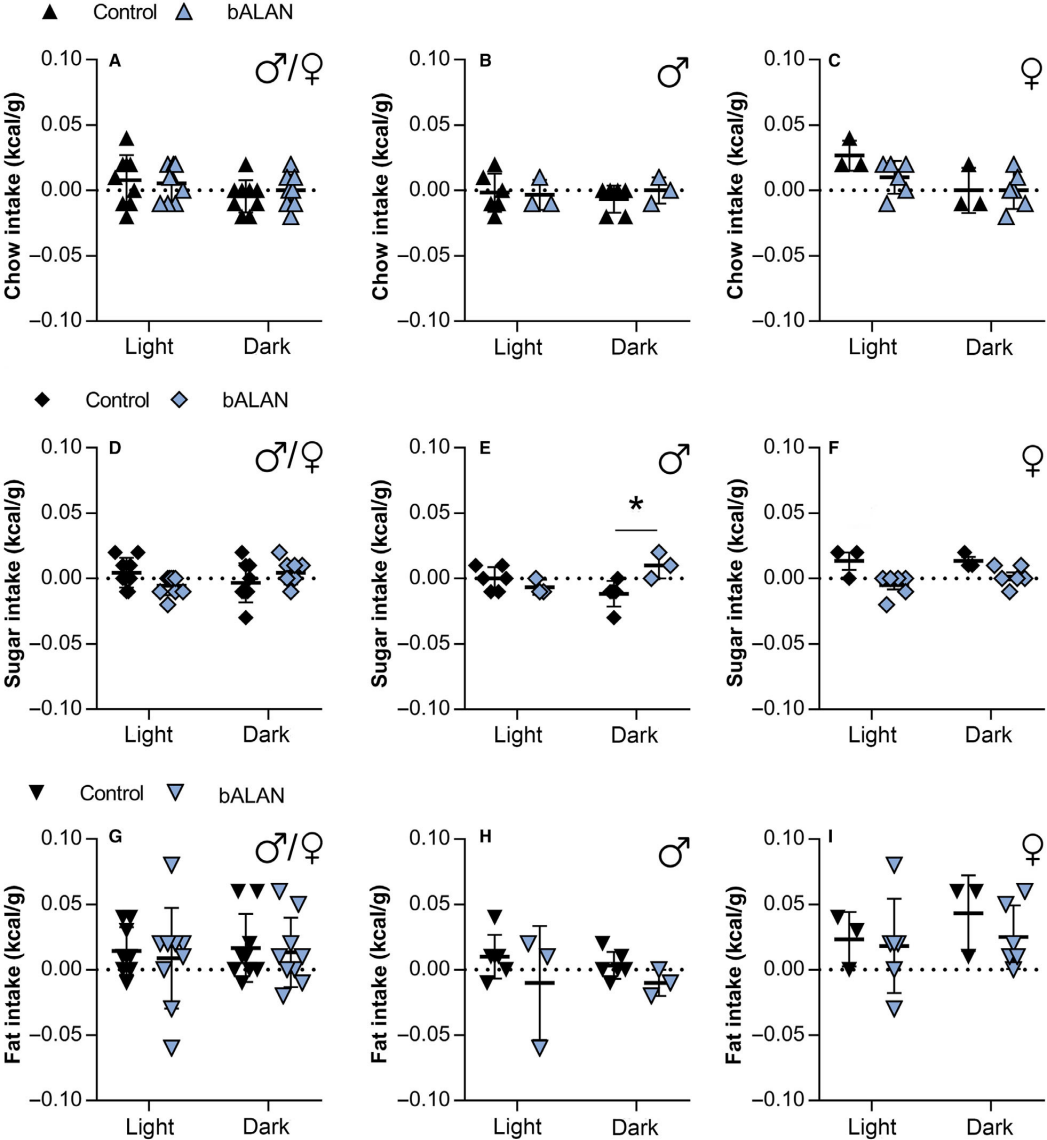
In HFHS‐fed *Arvicanthis*, bALAN exposure increases the sugar intake in male grass rats during the dark phase. bALAN exposure from ZT14‐15 did not affect the consumption of chow food in all HFHS fed animals (A) nor in males (B) neither in females (C). The bALAN exposure did not affect either the sugar intake of all animals (D) but caused an increase in the sugar consumption during the dark phase in males (E; *Post‐hoc, *P* = 0.008), but not in females (F). bALAN exposure did not changed the amount of fat ingested in all animals (G), nor when analyzed separately in males (H) or females (I).

Nevertheless, in the HFHS‐fed group when the different components of the diet were analyzed separately (Fig. 8A–I), we found a significant difference for sugar consumption (*F*_(1,16)_ = 11.6, *P* = 0.004), suggesting that sugar intake was different between the dark‐ versus the bALAN‐exposed animals. Moreover, when we analyze sugar intake with respect to sex, a significant interaction (treatment × phase) effect was observed in males (*F*_(1,7)_ = 11.8, *P* = 0.011, post‐hoc, *P* = 0.008), indicating that bALAN‐exposed male *Arvicanthis* drink more sugar at the dark phase than their respective controls (dark‐exposed animals; Fig. 8E). In females, the ANOVA indicated a significant difference for the factor treatment (dark vs. bALAN) in sugar consumption (*F*_(1,7)_ = 9.0, *P* = 0.02; Fig. 8F); however, no differences were found for the factor phase (light vs. dark; *F*_(1,7)_ = 1.1, *P* = 0.3) or in the interaction between factors (*F*_(1,7)_ = 1.1, *P* = 0.3; Fig. 8F).

### Acute effects of blue light on blood glucose and hormones

**Figure 9:**
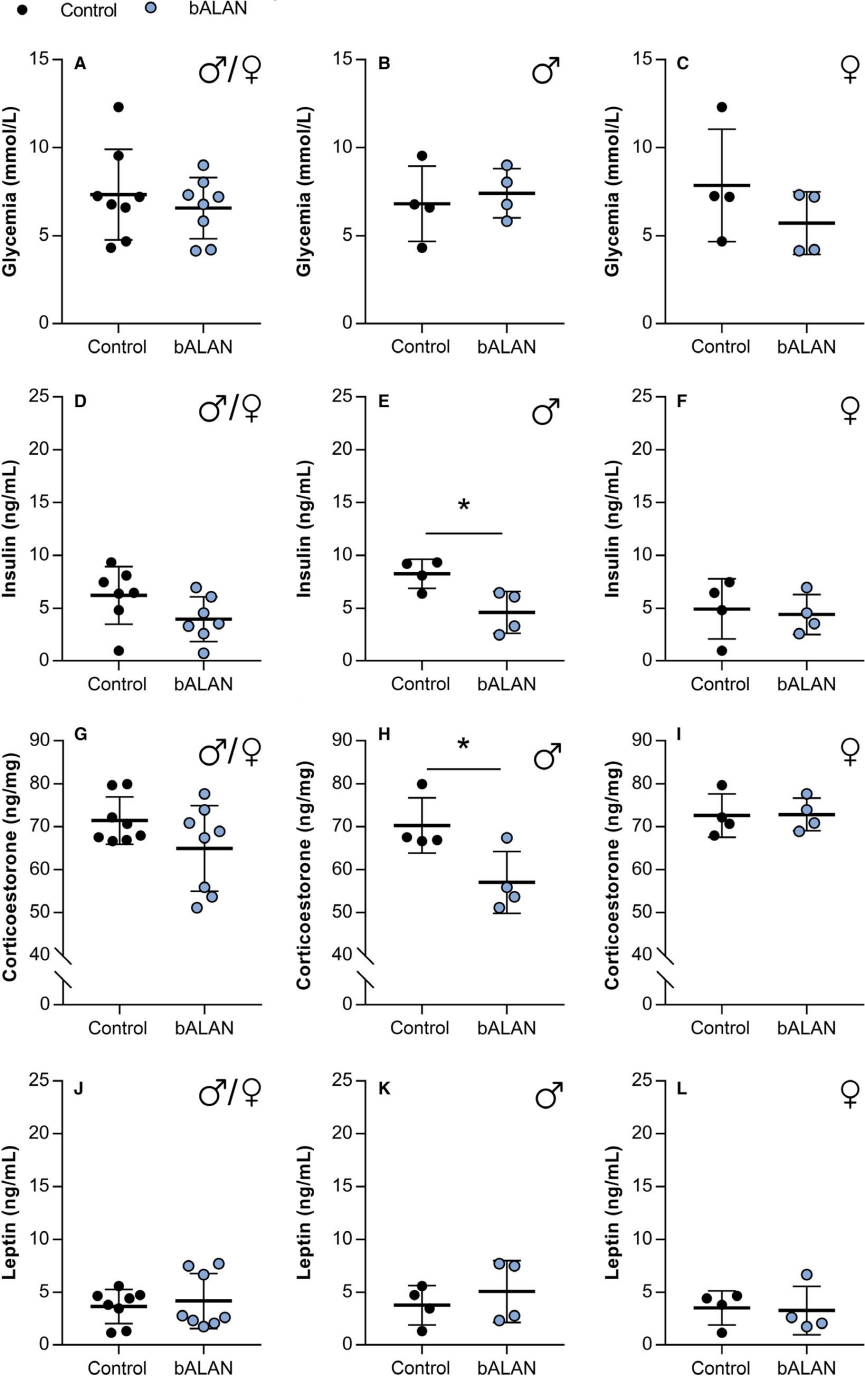
bALAN exposure decreased plasma insulin and corticosterone in male *Arvicanthis ansorgei* on a regular chow diet. bALAN exposure from ZT14‐15 in grass rats does not affect glycemia immediately after the light pulse in all animals (A), neither separately in males (B) or females (C). The bALAN exposure did not change the levels of plasma insulin in all animals (D), whereas it reduced the plasma insulin in male *Arvicanthis* (E; *t‐test, *P* = 0.02), but not in females (F). bALAN exposure did not changed the plasma corticosterone concentrations in all animals (G); however, lower levels of corticosterone were observed in bALAN‐exposed males (H; *t‐test, *P* = 0.03), but not females (I), when compared to the levels of cortiscosterone in control dark‐exposed *Arvicanthis*. The bALAN exposure pulse did not change plasmatic leptin levels in all animals (J), neither when analyzed separately for males (K) and females (L).

**Figure 10:**
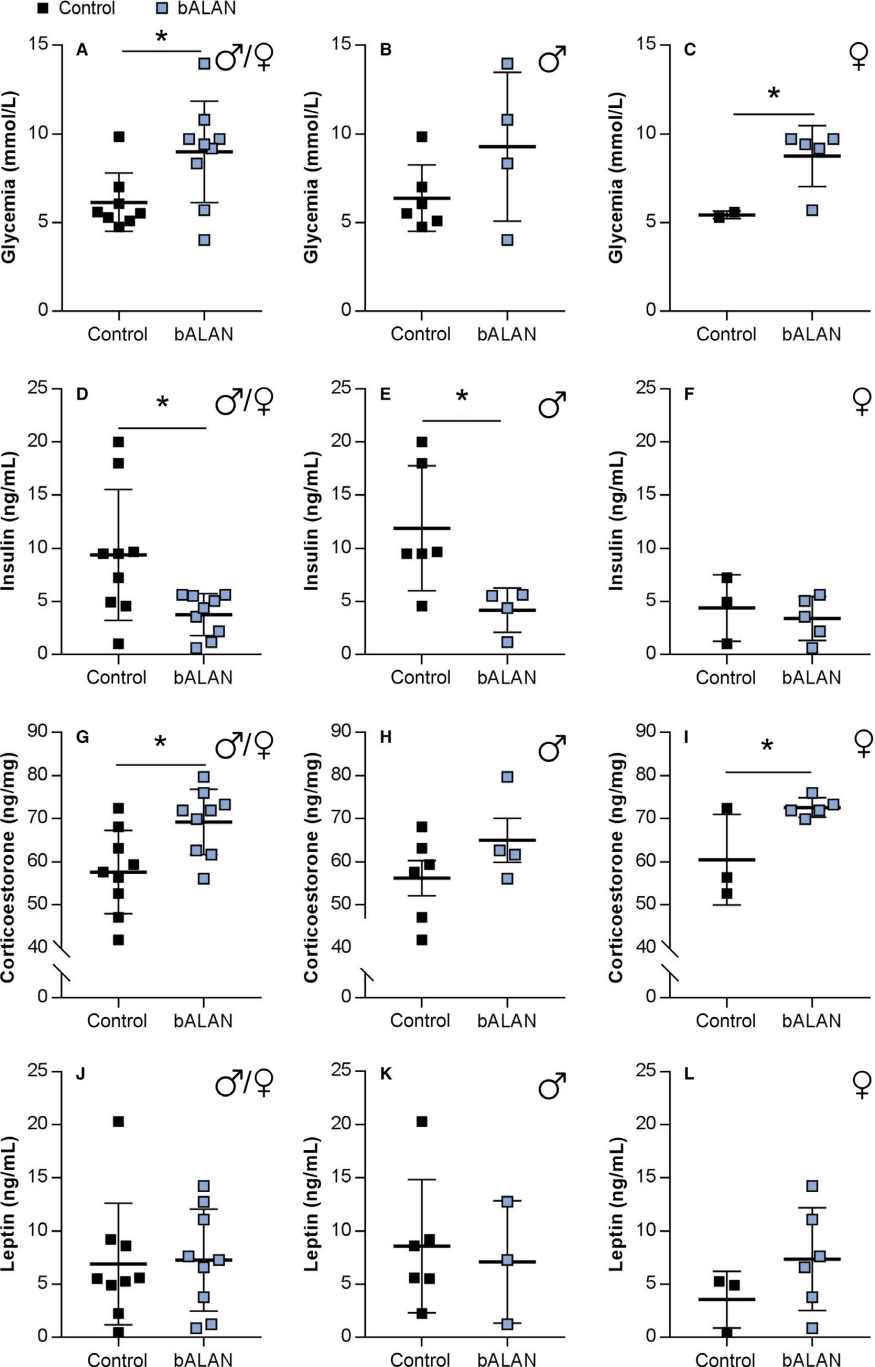
bALAN exposure increased glycemia, decreased insulin, and increased corticosterone in *Arvicanthis ansorgei* on a HFHS diet. bALAN from ZT14‐15 in grass rats increased blood sugar in all animals immediately after the light pulse (A; *t‐test, *P* = 0.026). Nevertheless, it did not cause blood glucose changes in males (B), whereas a significant increase was observed in females (C; *t‐test, *P* = 0.04). The bALAN exposure decreased the levels of plasma insulin in all animals (D; *t‐test, *P* = 0.01), with a significant reduction also observed in the plasma insulin of males (E; *t‐test, *P* = 0.03), but not in females *Arvicanthis* (F). bALAN exposure increased the amount of plasma corticosterone in all animals (G; *P* = 0.01). This effect was not significant separately in males (H), but it was significant in females (I; *t‐test, *P* = 0.03). The bALAN exposure did not change the plasmatic leptin levels in all animals (J), neither when analyzed separately in males (K) and females (L).

Plasma glucose levels were also measured after the third 1h bALAN exposure (Figs. 9A–C and 10A–C). No significant differences were found between dark controls versus bALAN‐exposed animals fed with a chow diet (*t*_(14)_ = 0.7, *P* = 0.4; Fig. 9A) neither in males (*t*_(6)_ = 0.4, *P* = 0.6; Fig. 9B) or females (*t*_(6)_ = 1.1, *P* = 0.2; Fig. 9C). On the other hand, in HFHS‐fed animals, increased blood glucose was observed after animals had been exposed to bALAN (*t*_(15)_ = 2.4, *P* = 0.02; Fig. 10A). When analyzed by sex, the hyperglycemic effect of the bALAN exposure was significant only in females (*t*_(5)_ = 2.5, *P* = 0.04; Fig. 10C), although a nonsignificant tendency of hyperglycemia was also observed in males (*t*_(8)_ = 1.5, *P* = 0.1; Fig. 10B).

After the third exposure to 1 h of bALAN exposure, in the chow‐fed group, no significant differences were detected in insulin levels when both sexes were analyzed together (*t*_(12)_ = 1.7, *P* = 0.1; Fig. 9D). However, in the group fed with the HFHS diet, animals exposed to bALAN exhibited significantly lower plasma insulin levels (*t*_(16)_ = 2.6, *P* = 0.01; Fig. 10D). When analyzed by sex, a significant reduction in plasma insulin was observed in bALAN‐exposed males, but not females animals, either fed chow (*t*_(6)_ = 3.0, *P* = 0.02; Fig. 9E) or the HFHS diet (*t*_(8)_ = 2.4, *P* = 0.03; Fig. 10E).

Levels of plasma corticosterone were not different in chow‐fed animals (*t*_(14)_ = 1.1, *P* = 0.12; Fig. 9G), but under HFHS diet significant higher levels of corticosterone were observed after the exposure to bALAN (*t*_(16)_ = 2.8, *P* = 0.01; Fig. 10G). The corticosterone analysis performed by sex showed higher levels of corticosterone only in HFHS‐fed females when exposed to bALAN (*t*_(6)_ = 2.6, *P* = 0.03; Fig. 10I), but lower levels in males on chow‐diet (Fig. 9H; *t*_(6)_ = 2.7, *P* = 0.03).

Lastly, no differences in plasma leptin levels were found due to the bALAN exposure in animals fed with the chow (*t*_(14)_ = 0.4, *P* = 0.6; Fig. 9J) or the HFHS diet (*t*_(16)_ = 0.1, *P* = 0.8; Fig. 10J). HFHS‐fed animals (Fig. 10J–L) had slightly higher leptin levels than the chow‐fed *Arvicanthis* (Fig. 9J–L), but this difference did not reach statistical significance (Dark controls, *t*_(15)_ = 1.5, *P* = 0.1; bALAN‐exposed, *t*_(15)_ = 1.6, *P* = 0.1).

## Discussion

To the best of our knowledge, this is the first study showing the effects of acute bALAN exposure on glucose metabolism and food intake in the diurnal rodent *Arvicanthis ansorgei,* fed either a chow or a HFHS diet*.* Importantly, the effects of bALAN on blood glucose and plasma insulin concentrations, as well as increased sugar intake were significant only in males*,* indicating a sex‐dependent mechanism. The use of a diurnal rodent exposed to a high‐fat high‐sugar free choice diet more resembles light‐exposure and feeding conditions in humans, than those in nocturnal rats or mice, and thus provides a better model for translational studies. Altogether, our results indicate that bALAN impairs glucose metabolism, especially in males, which in the long run could lead to the development of diabetes.

Previously, the HFHS diet has been used successfully to induce obesity in rats (La Fleur et al. 2010, 2014) and mice (Blancas‐Velazquez, la Fleur and Mendoza 2017), and here we used this dietary paradigm for the first time in the diurnal rodent *Arvicanthis ansorgei*. We did not observe a diet‐induced increase in body weight in either sex, but although adiposity was not measured, plasma leptin was slightly elevated in HFHS animals. Moreover we did observe marked glucose intolerance in both HFHS‐fed males and females *Arvicanthis* like previously described in nocturnal rats and mice (La Fleur et al. 2010; Blancas‐Velazquez et al. 2017). After nocturnal exposure to blue light, increased glucose levels were observed in male *Arvicanthis* regardless of the type of diet, together with decreased plasma insulin levels. These results further stress the negative effects of nocturnal light on glucose tolerance, putatively by inhibiting insulin release, as previously also described in rats (Opperhuizen et al. 2017) and in healthy and T2D adults (Cheung et al. 2016; Versteeg et al. 2017). It has been described before how light during the dark phase via the SCN can increase the sympathetic activity and decrease the parasympathetic activity of autonomic nerves that reach peripheral organs like the liver, the pancreas and the adrenal gland (Niijima et al. 1992, 1993; Ishida et al. 2005); this would lead to an increase in the hepatic gluconeogenesis, glycogenolysis, and a decrease in the release of insulin from the beta cells which can explain our current results in the glucose tolerance test.

Contrary to reports in nocturnal rats (La Fleur et al. 2014), a HFHS diet did not induce snacking behavior during the inactive phase in grass rats. However, exposure to the HFHS diet did to some extent reduce locomotor activity at the light phase in both sexes. Interestingly, although males did not change their food intake rhythm or increase their total caloric intake when exposed to a HFHS diet, an increased preference for fat consumption was observed at the middle of the light phase (ZT5) in some animals. Females developed hyperphagia during their active phase when exposed to the palatable diet, with a ~50% increase in total caloric intake compared to chow‐fed females. Previous studies in Wistar rats have shown that when rats are allowed to self‐select the macronutrient composition of their meals, a high‐protein high‐fat composition is chosen by both sexes. Especially males have a higher fat intake after 6 weeks of age, mainly during their active phase (i.e., the dark period) (Jean et al. 2002), in agreement with the preference for fat at ZT5 in the male *Arvicanthis*. Earlier work from other groups has shown that female rats have a higher preference for diets with a high content of carbohydrates (Yang et al. 2017), which would be in agreement with the hyperphagia observed in female *Arvicanthis*, coming mainly from the higher intake of sugar.

Animal and human studies providing evidence of the metabolic effects of environmental light have been reviewed extensively (Cho et al. 2015; Versteeg et al. 2016). In humans, increases in obesity and diabetes match increased artificial light exposure (Fonken and Nelson 2014) and it has been demonstrated that evening bright light increases appetite (AlBreiki et al. 2015). Additionally, in a human study which aimed to assess the effect of bALAN exposure on the control of sleep/wakefulness and energy metabolism, it was reported that energy expenditure, oxygen consumption, carbon dioxide production and the thermic effect of breakfast were significantly lower in subjects who received bALAN compared to no light exposure (Kayaba et al. 2014). Opposed to studies using chronic exposure to dim light at night (Fonken et al. 2010; Cissé et al. 2017), we did not observe an increase in the total amount of calories ingested by animals as an acute effect of the light pulse. However, when we analyzed separately each component of the diet, we found a significant increased sugar intake during the dark phase in male animals after a 1 h of bALAN exposure, suggesting that blue light may affect specific brain sites regulating palatable food intake, in a sex‐dependent manner.

Our observations in this experiment indicate that sex is an important variable when accounting for light effects not only on glucose metabolism but also on food intake. Although possible mechanisms responsible for sex differences regarding light perception in rodents are virtually unknown, a study in humans demonstrated that compared to women, men show a stronger response to blue‐enriched light in the evening even at very low intensity (Chellappa et al. 2017). Clearly, further research is needed to elucidate the physiology behind these differences. Moreover, animal studies have reported how female animals are protected from circadian metabolic disruptions by high‐fat feeding due to the ovarian hormones (Palmisano et al. 2017), which may be a reason why we did not observe the same detrimental effects with the HFHS diet and the bALAN in females.

While the central projections of the ipRGCs have not been described in *Arvichanthis ansorgei,* previous work has been done in diurnal rodents from the same genus: *Arvicanthis niloticus* (Langel et al. 2015). The results of that study pointed out that the main characteristics of these cells are fundamentally the same as those described in nocturnal rodents (Hannibal and Fahrenkrug 2004; Hattar et al. 2006; Reifler et al. 2015). Thus, among the areas that are innervated by the ipRGCs are the SCN, the intergeniculate leaflet (IGL), and presumably the lateral hypothalamus (LH). In previous work from another group, the acute effects of light on the brain of *Arvicanthis* have been reported (Shuboni et al. 2015), showing a significant number of c‐Fos activated cells in the IGL, the LH, and the lateral habenula (LHb), or the perihabenular nucleus (PHb) in mice (Fernandez et al. 2018). It has been previously shown that the geniculohypothalamic tract (which includes the IGL), actively modulates SCN responses to retinal input (Hanna et al. 2017) and might modulate metabolic signals to the SCN (Saderi et al. 2013), and reward and metabolic signals of feeding to the LHb (Huang et al. 2019). Also, it is widely known that the LH and the LHb are deeply involved in the control of metabolism, food intake and motivational processes (Kalsbeek et al. 2010; Salaberry and Mendoza 2016), which could explain our findings in this study.

In previous studies from our group, we reported decreased glucose tolerance without an insulin response when Wistar rats were exposed to a two hour bright or green light pulse from ZT14‐16. However, this was not observed with blue light. Additionally, in that same study increased insulin responses with only small effects on glucose levels were observed when ALAN was administered close to the end of the dark phase, suggesting that not only wavelength but also time‐of‐day defines the effects of ALAN on glucose metabolism, probably through various mechanisms (Opperhuizen et al. 2017). However, there are several differences in the experimental set‐up, such as the wavelength of the lights used, length of the light exposure, glucose administration route, blood sampling method, and the diurnality of the *Arvicanthis* versus the nocturnal nature of the rats that make it difficult to compare both experiments. Further studies with bALAN and diurnal species are needed to evaluate more in‐depth the putative effects of blue light on glucose metabolism and food intake in humans.

To conclude, we showed that in the diurnal rodent *Arvicanthis ansorgei* acute exposure to bALAN causes glucose intolerance, reduced insulin secretion and increased consumption of sugar, especially in males. Whether this effect is dependent on the activation of melanopsin in the ipRGCs and thus its projections to different parts of the brain, remains to be investigated. Nevertheless, our present results in this diurnal rodent and previous results in rats (Opperhuizen et al. 2017) and humans (Cheung et al. 2016; Versteeg et al. 2017) are sufficient to raise the awareness about the deleterious effects of light at night, especially short‐wavelength coming from light‐emitting diodes based products, on glucose metabolism and food intake.

## Conflict of Interest

None.
